# How do Autistic and Neurotypical Children’s Interests Influence their Accuracy During Novel Word Learning?

**DOI:** 10.1007/s10803-023-06066-8

**Published:** 2023-08-02

**Authors:** Charlotte Rothwell, Gert Westermann, Calum Hartley

**Affiliations:** Department of Psychology, Lancaster University, Lancaster, LA1 4YF UK

**Keywords:** Word learning, Autism, Interests, Attention, Referent selection, Retention

## Abstract

Word learning depends on attention – children must focus on the right things at the right times. However, autistic children often display restricted interests, limiting their intake of stimuli during word learning. This study investigates how category interests influence word learning in autism and neurotypical development. Autistic and neurotypical children matched on receptive vocabulary used a touch-screen computer to learn novel words associated with animals (high-interest stimuli) and objects (neutral-interest stimuli) via fast mapping. Response accuracy and speed were examined at referent selection, 5-minute retention, and 24-hour retention. Both groups identified meanings of novel words associated with unfamiliar animals and objects via mutual exclusivity with comparable accuracy. After 5 minutes, autistic children retained animal names with greater accuracy than neurotypical children. Autistic children showed a greater increase in their accuracy between 5-minute and 24-hour retention and outperformed neurotypical children across conditions after a night’s sleep. Across groups, 24-hour retention was predicted by number of target word repetitions heard at referent selection, indicating a relationship between fast mapping input and retention. However, autistic children were slower to respond correctly, particularly in the animal condition. For autistic children, superior word learning associated with high-interest stimuli was relatively short-term, as sleep appeared to consolidate their memory representations for neutral-interest stimuli. Although these results demonstrate that fundamental word learning mechanisms are not atypical in autism, slower response times may signal a speed-accuracy trade-off that could have implications for naturalistic language acquisition. Our findings also indicate favourable environmental conditions to scaffold word learning.

Word learning is one of the most important milestones in children’s cognitive development (Carpenter et al., 1998). While neurotypical children can map word-referent associations from 6-months (Friedrich & Friederici, 2011) and know the meanings of approximately 200 words before 2-years of age (Dale & Fenson, 1996), autism is often characterised by significant delays in receptive vocabulary development (Artis & Arunachalam, 2023; Kover et al., 2013). Recent studies investigating the causes of autistic children’s difficulties acquiring vocabulary have demonstrated that fundamental word learning mechanisms function and inter-relate in a manner that resembles neurotypical development (Carter & Hartley, 2021; Hartley et al., 2019, 2020). Thus, it may be that autistic children’s word learning difficulties can be attributed to attentional differences that affect their intake of visual and auditory input (Arunachalam & Luyster, 2018; Venker et al., 2018). Here, we directly test this theory by systematically investigating how autistic children’s interests in stimuli influence multiple word learning mechanisms. Throughout the manuscript we use identity-first language as this is often preferred by the autism community (e.g. Kenny et al., 2016).

When a child detects a novel word in speech, successful word learning is contingent on accurately identifying its intended meaning (referent selection; Vlach & Sandhofer, 2012). The child must then store the correct word-referent association in memory for later retrieval (retention; Gleitman, 1990). According to the ‘dynamic associative account,’ referent selection and retention utilise separate ‘fast mapping’ and ‘slow learning’ mechanisms that operate on different timescales (McMurray et al., 2012).

Fast mapping occurs when children overcome the challenge of referential ambiguity (there are often multiple potential targets for a newly heard word; Markman, 1989) by correctly inferring meaning from linguistic and environmental cues (Carey & Bartlett, 1978). For example, by 2 years, neurotypical children map new word-referent associations on the basis that each word has only a single referent (they employ the principle of ‘mutual exclusivity (ME);’ Markman, 1989). Children’s use of ME is commonly tested by presenting an unfamiliar object amongst familiar objects and asking them to identify the referent of a novel word. As the familiar objects already have known labels, neurotypical children deduce that the unfamiliar object must be the referent for the novel word.

Although referent selection is an important first step towards vocabulary acquisition, children are considered to have ‘learnt’ a new word only when they can retrieve its meaning after a delay (Gleitman, 1990). Crucially, accurate referent selection does *not* guarantee retention; Horst and Samuelson (2008) demonstrated that neurotypical toddlers who perform at ceiling on a fast-mapping task often fail to retain novel words after five minutes (also see Bion et al., 2013). While referent selection represents a process of attentional narrowing, retention is underpinned by basic associative learning mechanisms that gradually strengthen as statistical input increases (Hartley et al., 2020; McMurray et al., 2012). Newly formed word-referent associations are also strengthened by sleep. School-aged neurotypical children’s novel word retention significantly improves after a night’s sleep (Brown et al., 2012), and preschool children who nap shortly after exposure to novel words are more likely to retain their meanings (Williams & Horst, 2014). These effects are explained by ‘active system consolidation theory,’ which proposes that sleep enhances retention by reactivating recently encoded word-referent representations, facilitating their integration into memory networks by strengthening synaptic connections (Diekelmann & Born, 2010).

Importantly, children’s word learning and attention are fundamentally inter-related. During fast mapping, children must focus their attention on a novel word’s intended referent while excluding non-target competitors (Twomey et al., 2016). This requires children to navigate their attention across multiple components of the learning environment *and* coordinate their attention to corresponding audio-visual stimuli during naming events (Samuelson et al., 2017). Ackermann et al. (2020) recently reported that neurotypical 30-month-olds find it easier to learn names for novel referents belonging to categories they are particularly interested in, such as animals. These findings suggest that heightened attention to interesting objects increases children’s focus, which in turn benefits their encoding of word-referent representations.

Early studies investigating autistic children’s referent selection identified reduced sensitivity to social-pragmatic cues as a potential cause of their language learning difficulties (e.g. Baron-Cohen et al., 1997; Preissler & Carey, 2005). However, a plethora of recent studies have demonstrated that autistic children with varying language abilities can successfully utilise social cues to inform accurate referent selection (e.g. Luyster & Lord, 2009; McGregor et al., 2013). Furthermore, autistic children – including those with delayed receptive vocabulary development – can accurately identify novel word meanings via lexical heuristics such as ME (Preissler & Carey, 2005).

In contrast to referent selection, few studies have investigated retention of newly learned words in autistic children with delayed language development. In two recent exceptions, Hartley et al. (2019, 2020) investigated the relationship between identification and retention of novel word meanings and explored how these processes are influenced by attentional cues. In their 2019 paper, language-delayed autistic children and neurotypical children matched on receptive vocabulary identified the names of novel objects in a ME-based fast-mapping task. After a 5-minute delay, autistic children responded at least as accurately as neurotypical children on a retention test. In Hartley et al. (2020), similar samples disambiguated word meanings by tracking statistical word-object co-occurrences with equivalent accuracy and the groups did not differ on retention tests. However, autistic children were significantly slower to indicate correct referents under both cued and non-cued learning conditions. These findings suggest that fundamental mechanisms supporting word learning, and the relationships between them, may not be qualitatively atypical in language-delayed autistic children. Rather, differences in response time may indicate that autism impacts the speed at which children process stimuli during word learning (Arunachalam & Luyster, 2018; Tenenbaum et al., 2017).

Whereas neurotypical children can flexibly navigate attention across their environment, many autistic children have difficulties allocating sustained/selective attention and shifting focus between stimuli (Noterdaeme et al., 2002). These differences in attention have been linked to domain-general deficits in executive functioning (Ozonoff et al., 2004), which in turn have been implicated as a potential cause of diagnosis-defining restricted and repetitive behaviours and interests (RRBIs; Richler et al., 2007). RRBIs result in children focusing intensely and repeatedly on very specific interests and activities in their daily lives. Such is the intensity of their RRBIs, many autistic children experience difficulty disengaging from preferred stimulus categories and may be reluctant to attend to stimuli that they find less interesting (Leekam et al., 2011). Since environmental input is carefully selected and restricted by the child’s interests, attentional focal points are narrowed (Elsabbagh et al., 2009) and sensitivity to valuable information in the environment may be suppressed (McGregor et al., 2013).

During word learning, restrictive attentional behaviours may prevent autistic children from attending to all stimuli in an array (Hartley et al., 2019). Many autistic children experience ‘sticky’ attentional fixations, and their focus is often captured by salient perceptual features to an atypical degree (Pierce et al., 2011). These attentional differences could have profound implications for language acquisition (Hilton et al., 2019; Hilton & Westermann, 2017). On one hand, if to-be-learned stimuli do not align with autistic children’s interests, reduced attention may result in weak or incorrect representations of word-referent relationships (e.g. Tenenbaum et al., 2017; Venker et al., 2018). Alternatively, if stimuli appeal to their interests, heightened attentional focus could lead to the formation of more robust word-referent relationships that are less susceptible to decay (e.g. Ackermann et al., 2020). Whilst some studies have explored how word learning in autism is influenced by external social and non-social attentional cues (e.g. Baron-Cohen et al., 1997; Preissler & Carey, 2005), no research to our knowledge has directly investigated how differences in internal preferential interests impact referent selection and retention in autism.

For the first time, the present study investigated how interests associated with specific categories of stimuli influence multiple word learning mechanisms in autistic children with delayed language development. Autistic and neurotypical children matched on receptive vocabulary identified the meanings of novel words in a computer-based ME referent selection task with two within-subjects conditions. In one condition, children learnt names for relatively interesting stimuli – unfamiliar animals (participants’ interest in animals was confirmed via a questionnaire). It is well-documented that children generally prefer animal stimuli over non-animal stimuli (Celani, 2002; Prothmann et al., 2009) and many autistic individuals are particularly fond of animals (Martin & Farnum, 2002). In another condition, children learnt names for unfamiliar objects – generic experimental stimuli that are less likely to align with children’s pre-existing interests. Retention of novel words was tested after 5 minutes and 24 hours. The retention tests following a 24-hour delay allowed us to investigate (a) the robustness of novel word representations relating to different categories, and (b) how sleep influences lexical consolidation in autistic children with concomitant language delay. Autism is often characterised by problematic sleep disorders, including bedtime resistance, sleep anxiety, difficulties falling asleep, and parasomnia (Díaz-Román et al., 2018; Souders et al., 2009). Given that sleep plays a critical role in protecting newly acquired declarative memories against decay in neurotypical development (Axelsson et al., 2018), such difficulties could impact autistic children’s consolidation of recently mapped word-referent associations. Although previous studies have identified benefits of sleep for autistic children’s lexical retention, these have exclusively recruited intellectually-able participants with high IQs who do not have language-learning difficulties (e.g. Fletcher et al., 2020; Henderson et al., 2014). Therefore, this study is the first to test whether overnight memory consolidation of new words differs for autistic children with delayed language development.

As numerous studies have shown that autistic and neurotypical children can accurately apply ME when fast mapping with generic novel objects (e.g. Carter & Hartley, 2021; Hartley et al., 2019), we did not expect any between-population or between-condition differences in accuracy during referent selection. However, we anticipated that differences in attention invested during referent selection may have consequences for retention. In particular, based on evidence for positive relationships between attentional focus and word learning (Ackermann et al., 2020; Axelsson et al., 2012; Bion et al., 2013), we predicted that children in both populations would retain names for unfamiliar animals with greater accuracy than names for unfamiliar objects. After 24 hours, we tentatively predicted that sleep-induced benefits for retention would be weaker for autistic children with delayed receptive vocabulary development than neurotypical controls. We also anticipated that autistic children would be slower to generate correct responses than neurotypical children across all word learning stages, potentially indicating differences in speed of processing audio-visual input (e.g. Hartley et al., 2020). Importantly, this research will advance theoretical understanding of word learning by revealing the influence of preferential biases to selective stimuli in both autism and neurotypical development.

## Methods

### Participants

Participants were 15 autistic children (13 males, 2 females; *M* age = 91.87 months; *SD =* 21.30) recruited from specialist schools, and 16 neurotypical children (6 males, 10 females; *M* age = 52.31 months; *SD =* 18.88) recruited from mainstream schools, nurseries, and Lancaster University BabyLab (see Table 1). All participants were monolingual, English was their native language, and had normal or corrected-to-normal colour vision. Autistic children were previously diagnosed by a qualified educational or clinical psychologist, using standardised instruments (i.e. Autism Diagnostic Observation Scale and Autism Diagnostic Interview – Revised; Lord et al., 1994, 2002) and expert judgement. Diagnoses were confirmed via the Childhood Autism Rating Scale 2 (CARS; autistic *M* = 34.70, *SD* = 10.23; neurotypical *M* = 16.78, *SD* = 2.56; Schopler et al., 2010). This measure was usually completed by class teachers, but for eight neurotypical children who were tested at our BabyLab due to COVID-19 restrictions, it was completed by caregivers. Autistic children were significantly older, *t*(29) = -5.48, *p* < .001, *d* = 1.97, and had significantly higher CARS scores, *t*(29) = -6.79, *p* < .001, *d* = 2.40, than the neurotypical children.

**Table 1:** Characteristics of autistic and neurotypical Participants (*SD* and Ranges in Parentheses)

Group	N	Gender	Chron. Age (*M* months)	BPVS age equiv. (*M* months)	Express. Lang. age equiv. (*M* months)	CARS raw score (*M*)	Leiter-3 raw score (*M*)	CTRS raw score (*M*)	RBQ raw score (*M*)	Animal Interest score (*M*)
NT^a^	16	6 males, 10 females	52.31 (18.88; 27–94)	60.31 (27.44; 36–118)	60.31 (22.76; 35–104)	16.78 (2.56; 15–24)	57.25 (17.93; 40–95)	12.25 (6.03; 2–26)	27.00 (5.80; 20–35)	23.31 (2.80; 19–29)
ASD^a^	15	13 males, 2 females	91.87 (21.30; 67–136)	53.27 (22.48; 24–97)	48.47 (27.70; 5–82)	34.70 (10.23; 20–52)	60.33 (15.57; 38–83)	17.27 (11.04; 5–36)	43.87 (8.37; 30–59)	23.93 (5.55; 17–34)
Group comparison *t*-test (*p*)			< .001	.44	.20	< .001	.64	.12	< .001	.69

^a^Note. NT: neurotypical; ASD: autism spectrum disorder; BPVS: British Picture Vocabulary Scale, CARS: Childhood Autism Rating Scale, CTRS: Conner’s Teacher Rating Scale, RBQ: Repetitive Behaviour Questionnaire

Groups did not significantly differ on receptive vocabulary as measured by the British Picture Vocabulary Scale 2 (BPVS; autistic *M* age equivalent = 53.27 months, *SD* = 22.48; neurotypical *M* age equivalent = 60.31, *SD* = 27.44; Dunn et al., 1997), *t*(29) = 0.78, *p* = .44. Receptive vocabulary was selected as our group matching criterion as it reflects children’s ability to learn word-referent relationships (Bion et al., 2013). Expressive vocabulary was measured using the Expressive Vocabulary Test 2 (EVT; Williams, 2007), or the expressive language module of the Mullen’s Scales of Early Learning (MSEL; Mullen, 1995) for children who scored below the baseline on the EVT. Autistic (*M* age equivalent = 48.47 months, *SD* = 27.70) and neurotypical children (*M* age equivalent = 60.31 months, *SD* = 22.76) did not significantly differ on expressive vocabulary, *t*(29) = 1.30, *p* = .20.

Children’s non-verbal intellectual abilities were measured using the Leiter-3 (Roid et al., 2013). The neurotypical group’s average non-verbal IQ score (*M* = 101.38, *SD* = 7.84) was significantly higher than the autistic group’s (*M* = 77.67, *SD* = 11.73), *t*(23) = 5.99, *p <* .001, *d* = 2.38. Scaled IQ scores could not be calculated for three neurotypical children as they were below the age of three years. However, the groups’ raw scores on the Leiter-3 did not significantly differ (autistic *M* = 60.33, *SD =* 15.57; neurotypical *M =* 57.25, *SD =* 17.93), *t*(26) = -0.48, *p* = .64, suggesting that their non-verbal cognitive abilities were similar at time of testing (when age was not considered). Three autistic children did not complete the Leiter-3 due to school closures during the COVID-19 pandemic, but they were retained in the study as they completed all other measures. To assess attentional behaviours, the Conner’s Teacher Rating Scale (CTRS-15; Pupura & Lonigan, 2009) was completed by children’s class teachers, or the caregivers of the eight neurotypical children who were tested in our BabyLab. The mean raw scores for the autistic children (*M* = 17.27, *SD =* 11.04) and neurotypical children (*M =* 12.25, *SD =* 6.03) did not significantly differ, *t*(29) = -1.58, *p* = .12. The Repetitive Behaviour Questionnaire was completed by participants’ caregivers to assess the extent of their restrictive and repetitive behaviours (RBQ; Leekam et al., 2007). Autistic children (*M =* 43.87, *SD =* 8.37) had significantly higher scores than neurotypical children (*M* = 27.00, *SD =* 5.80), *t*(29) = -6.56, *p* < .001, *d* = 2.34.

Finally, we designed a caregiver questionnaire to assess the extent to which children were interested in animals (min-max scores: 0–34; autistic *M* score = 23.93, *SD* = 5.55, neurotypical *M* score = 23.31, *SD* = 2.80; see Supplementary Materials). The purpose of this measure was to ensure that we recruited participants who were interested in animals, validating our categorisation of stimuli in the animal condition as ‘high-interest.’ The groups did not differ significantly on this measure, *t*(29) = -0.40, *p* = .69. One autistic child was excluded from the study due to their lack of interest in animals.

An additional four participants were excluded from the study; two neurotypical participants who were unable to complete the touch screen task, and two children who did not complete both experimental conditions (one autistic and one neurotypical child).

All procedures performed in this study involving human participants were in accordance with the ethical standards of institutional and national research committees. Informed consent was obtained from caregivers prior to children’s participation and a debrief was provided after participation.

### Materials

**Fig. 1:**
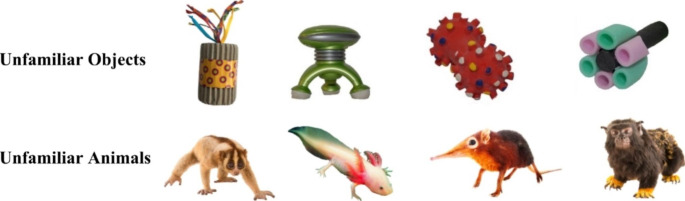
Sets of unfamiliar objects and animals used in the word learning task

The study was administered via a touch-screen computer running MATLAB. Audio stimuli for the word learning task included eight two-syllable unfamiliar words (manu, tanzer, boskot, virdex, toma, fiffin, chatten, modi) selected from the NOUN database (Horst & Hout, 2016) and other academic sources. Visual stimuli included high-resolution colour photographs of 4 unfamiliar objects, 4 unfamiliar animals (see Fig. 1), and 22 familiar objects, all presented on a grey background. All photographs were approximately 6cm^2^ and 500 × 500 pixels when displayed on the screen. Unfamiliar stimuli were selected on the basis that children would not know their linguistic labels. Familiar objects were selected on the basis that most children understand their linguistic labels by 16 months (Fenson et al., 1994). Pictures of six familiar objects were employed in warm-up trials (tree, door, light, slide, pram, top). Pictures of 16 familiar objects were presented during referent selection trials in the object condition and animal condition. These were divided into two sets and counterbalanced across conditions (1. bottle, hat, pillow, toothbrush, rock, balloon, truck, bath; 2. telephone, ball, chair, spoon, bed, window, fridge, towel). Familiar objects allocated to the two conditions were matched on mean comprehension age (13.5 months for both sets) and frequency of objects belonging to particular categories (e.g. toys, furniture). Familiar objects within each set were divided into pairs and presented alongside an unfamiliar object or animal in referent selection trials (depending on condition). In every trial type, three pictures were presented side-by-side. We ensured that names of stimuli presented together were phonologically distinct and their images clearly contrasted in shape and colour.

Stimuli names were recorded by a female speaker from the local area and presented through the computer’s integrated speakers. Audio files were recorded and edited using a Sony ECM-MS907 Digital Microphone and Audacity 2.2.2 software. Auditory stimuli were edited for timing and clarity, and the volume of all files was normalised. The carrier phrases (e.g. “Can you see the [label]?”, “Touch the [label]!”) and the labels (e.g. “tree”, “fiffin”) were edited separately, so they were all distinct files. However, when the MATLAB programme ran the experiment, the audio files were presented sequentially. This was to ensure that there were no differences in the carrier phrases that may offer a hint to children regarding the labels that were about to be presented. Three web cameras attached to the left, right, and centre of the computer were used to record participants’ visual attention and behaviour during the study, although these data are not reported in the present paper.

### Procedure

During recruitment, caregivers completed a questionnaire about their child’s interest in animals (see Supplementary Materials). Animals are a common interest of many autistic and neurotypical children (Martin & Farnum, 2002; Prothmann et al., 2009), and our objective was to explore how this interest would influence their relative performance in the two word learning conditions. Examples of questions included: ‘How much does your child like animals?’ (responses: 1 - they don’t mind animals, 2 - they like animals a little, 3 - they like animals a lot, 4 - they really, really like animals) and ‘How much does your child enjoy watching television programmes, videos, and films involving realistic animals?’ (responses: 1 - they don’t particularly enjoy it, 2 - they enjoy it a little, 3 - they enjoy it a lot, 4 - they really, really enjoy it).

**Fig. 2:**
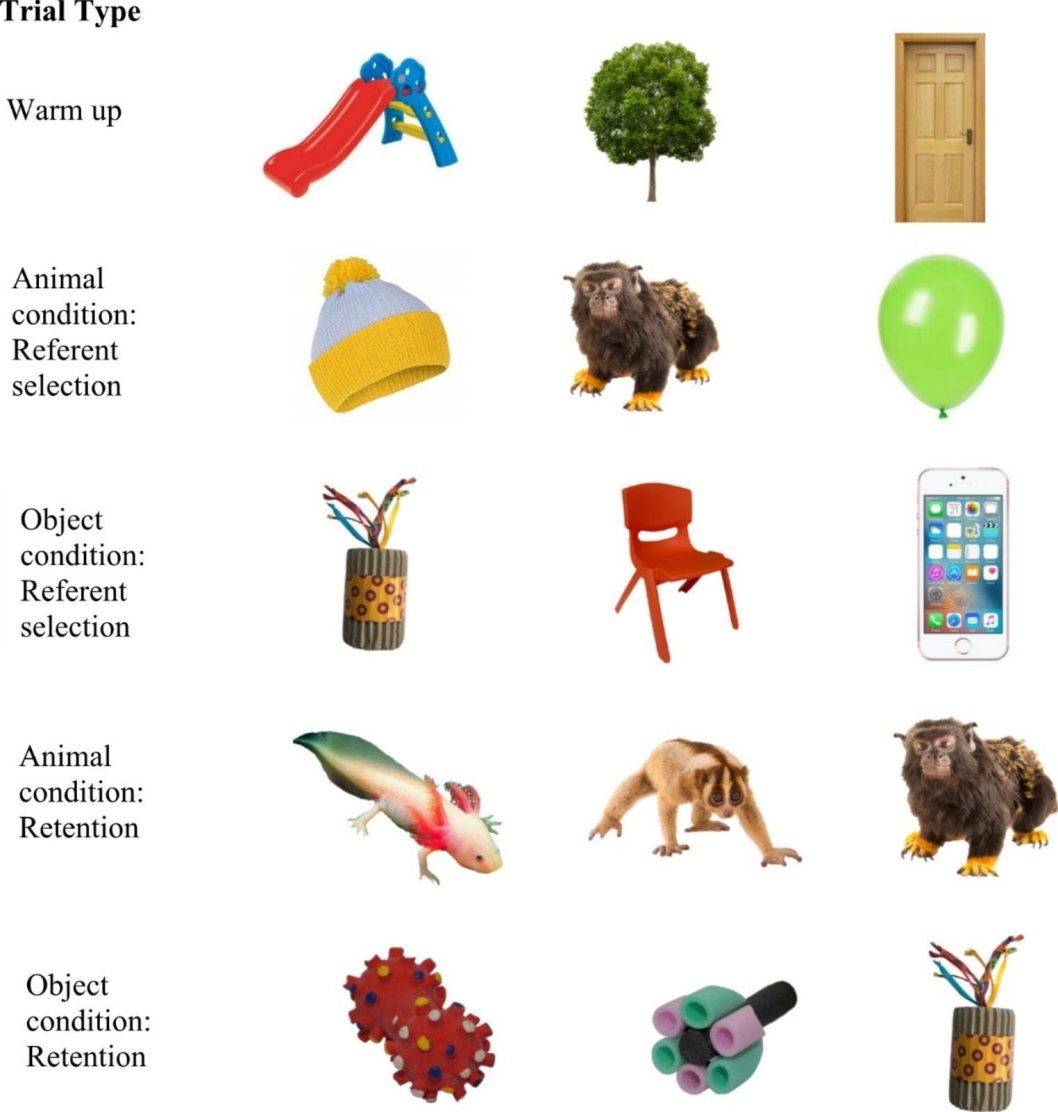
Examples of trial types in the word learning task

Participants were tested individually in their own school or nursery, or our BabyLab, and were accompanied by a familiar adult when required. Children were assessed using the Leiter-3, BPVS, and EVT or MSEL by the researcher over multiple sessions on different days. Children completed two within-subjects conditions of the word learning task – novel animals and novel objects – administered on different days (average of six days apart, order counterbalanced). The word learning task was delivered via a touch-screen computer. Children were seated approximately 50–70 cm away from the screen on a height-adjustable chair. The word learning task consisted of the following stages, presented in a fixed order: (1) Warm-up trials, (2) Referent selection trials, (3) Five-minute delay, (4) Retention trials, (5) 24-hour delay, (6) Retention trials (see Fig. 2). The experimenter sat quietly while the participant was engaged in tasks and offered verbal praise for attention and good behaviour.

#### Warm up Trials

Before the study started, children were presented with a cartoon image of a hand that appeared in each of three touch-screen panels, one by one. To encourage children to feel comfortable touching the screen, the experimenter asked them to “Put their hand on the picture.” Then, children completed three warm-up trials. Children were instructed to “Put your hand on the picture that the computer asks you to.” During warm-up trials, children were presented with images of three familiar objects in the left, middle, and right sections of the computer screen. After 2 s, participants heard “Look, ‘*2 s gap*’ [label]!”, ‘*1 s gap*’, “Can you see the [label]?”, ‘*1 s gap*’, “Touch the [label]!”. Children then had 12 s to respond. The same instructions played up to six times if children did not respond. Responses were accepted only after the first label utterance, preventing children from skipping through trials without hearing the requested labels. Consequently, children who took longer to respond heard more repetitions of the label (this factor is examined in our analyses). Children received feedback when they made their selection; either audio praise if they responded accurately (e.g. “Well done, you touched the [label]!”) or corrective feedback if they responded inaccurately (“Actually, this is the [label]. Can you touch the [label]?”). Following inaccurate responses, the correct referent was highlighted by a green border and children could retry up to five times. The location and order of requested objects were counterbalanced across participants. Neurotypical (*M* = 0.95) and autistic (*M* = 0.90) children did not significantly differ in their response accuracy on warm-up trials, *t*(29) = 0.98, *p* = .33. This demonstrates that the groups were similar in their understanding of familiar labels and task requirements.

#### Referent Selection Trials

After the warm-up trials, children completed eight referent selection trials. These followed exactly the same format, except children did not receive feedback following their responses. Four novel words were taught via a fast-mapping paradigm based on Horst and Samuelson (2008). Children viewed four sets of pictures (each containing one unfamiliar picture and two familiar pictures). Each set was presented twice; on one trial the novel picture was requested (novel name trial: “Look, modi! Can you see the modi? Touch the modi!”), and on another trial a familiar picture was requested (familiar name trial: “Look, ball! Can you see the ball? Touch the ball!”). Familiar trials were included to detect whether participants’ responses were biased by a preference for novelty and to encourage them to examine every item in each array (accurate fast mapping requires children to attend to familiar competitors in order to exclude them as referents for a novel word; Halberda, 2003). Novel name trials promoted active learning of new word-referent pairings; since participants already knew labels for the familiar pictures, they could identify the referent of the novel label by applying the ME principle. During this stage, familiar stimuli were always objects, and novel stimuli were either animals (high-interest) or objects (neutral-interest), condition dependent.

Trial order was pseudo-randomised with the constraints that the same set of pictures, or the same trial type (familiar name or novel name), was not presented on more than two trials sequentially. Positioning of stimuli on the screen (left, middle, right) was pseudo-randomised across trials with the constraint that the target did not appear in the same location more than twice consecutively. The eight novel words were divided into two sets (1. manu, tanzer, boskot, virdex; 2. toma, fiffin, chatten, modi) and were counterbalanced across conditions. Novel words were pseudo-randomly allocated to novel referents, so different novel words represented different novel referents across participants. Familiar stimuli were divided into two sets of eight to obtain a degree of control, but these were also counterbalanced across conditions.

#### 5-minute Delay

Immediately after referent selection, children engaged in an unrelated task for five minutes (e.g. colouring or building with blocks). None of the familiar or unfamiliar experimental stimuli were visible during this stage.

#### Retention Trials

Following the five-minute delay, children completed one warm-up trial to re-engage their attention (exactly as described above). Eight retention trials immediately followed; three novel stimuli that were named during the referent selection trials were presented on screen in a row (left, centre, right) and children were asked to identify one (see Fig. 2 for an illustration of each trial type). Children’s memory for each word-referent pairing taught during referent selection was tested on two retention trials. These trials enabled us to assess whether children’s retention of newly mapped word-referent associations differed between high-interest (animal) and neutral-interest (object) stimuli. Trial order was pseudo-randomised, ensuring that the same set of stimuli was never presented on more than two trials sequentially. Positioning of stimuli on the screen (left, middle, right) was pseudo-randomised across trials with the constraint that the target did not appear in the same location more than twice consecutively. Each picture was a target on two trials and a foil on four trials.

#### 24-hour Retention Trials

After a 24-hour delay, children completed a second block of eight retention trials. Due to practical constraints, not all children experienced exactly a 24-hour delay (*M* delay = 23.8 h, range: 20.5–25.6 h). These retention trials were preceded by three warm-up trials (as described above) to remind children of the task requirements and how to respond. The 24-hour retention trials were identical to the 5-minute retention trials with the exception that stimuli were presented in different orders and combinations.

## Results

Accuracy and response time data were analysed via mixed-effects models using the glmer and lmer functions from the lme4 package in R (Bates et al., 2015). Population was contrast coded as -0.5 (neurotypical) and 0.5 (autistic). Condition was coded as -0.5 (novel object) and 0.5 (novel animal). Trial type was coded as -0.5 (familiar) and 0.5 (novel). By-word referent selection accuracy was coded as -0.5 (incorrect) and 0.5 (correct) when included as a fixed effect in retention accuracy analyses. Total accuracy at referent selection for novel trials was coded as 0–4. Number of repetitions of the target word heard at referent selection was coded as 1–7 (autistic *M* = 2.27, *SD* = 1.00; neurotypical *M* = 1.84, *SD* = 0.89). Total accuracy at 5-minute retention was coded as 0–8. Trial-level accuracy as a dependent measure was coded as 1 (correct) or 0 (incorrect) for all analyses.

The likelihood of children responding correctly by chance on each trial was 33%. All models were built up sequentially, adding fixed effects individually and comparing each model with the previous best-fitting model using log-likelihood tests. Each analysis started with a baseline model containing by-participant and by-word random intercepts, with a random slope of condition x trial type per participant (referent selection), or condition per participant (retention phases). If some models in a sequence were singular fitting or failed to converge, random effects were simplified until all models in the sequence successfully converged. Only final models are reported; please refer to Supplementary Materials for full details of model building sequences and analyses of individual differences.

### Referent Selection Accuracy

**Fig. 3:**
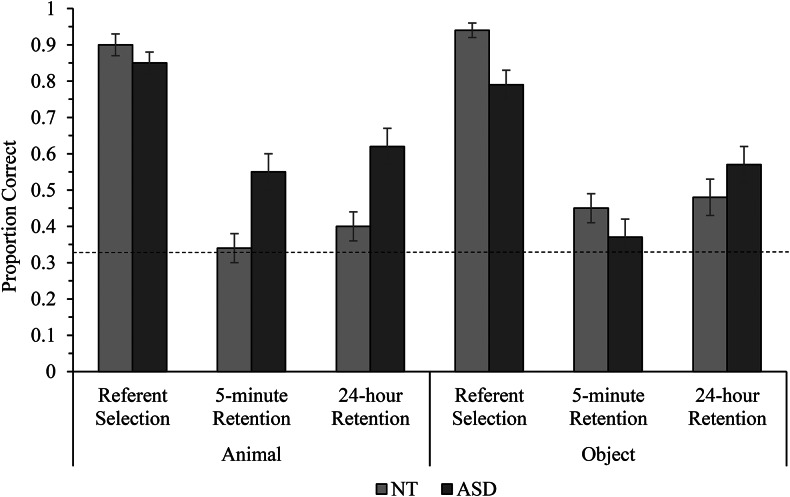
Mean referent selection, 5-minute retention, and 24-hour retention trial accuracy for neurotypical (NT) and autistic children (ASD), error bars show ± 1 *SE*

Referent selection accuracy was analysed via generalised linear mixed-effects models testing the effects of population, condition, and trial type. Five trials were excluded from autistic participants who simultaneously responded to different locations with their head and hands. This analysis contained 491 data points. Descriptive statistics for referent selection accuracy are presented in Fig. 3.

The best fitting model included a fixed effect of trial type (*z* = -5.19, *p <* .001; see Table 2) indicating that autistic and neurotypical children responded with significantly greater accuracy on familiar trials than novel trials. However, it is noteworthy that both groups responded well above chance on novel trials with object and animal targets (neurotypical children, animal condition *M* = 0.86, neurotypical children, object condition *M* = 0.88; autistic children, animal condition *M* = 0.81, autistic children, object condition *M* = 0.73), demonstrating their effective use of mutual exclusivity.

**Table 2:** Summaries of the fixed effects in the final generalised and linear mixed-effects models (log odds) of children’s accuracy on referent selection trials, and response times on correctly-answered referent selection trials

	**Fixed effects**	**Estimated** **coefficient**	**Std. error**	***z***	**Pr(> |z|)**
Accuracy	(Intercept)	3.42	0.48	7.13	< .001
	Trial Type	-2.59	0.50	-5.19	< .001
		**AIC**	**BIC**	**logLik**	**deviance**
		326.1	380.7	-150.1	300.1
	**Fixed effects**	**Estimated coefficient**	**Std. error**	***t***	**Pr(> |t|)**
Response Times	(Intercept)	3.41	0.33	10.35	< .001
	Population	1.44	0.66	2.19	.037
	Condition	0.07	0.19	0.38	.70
	Trial Type	0.85	0.19	4.40	< .001
	Population x Condition	1.03	0.39	2.65	.008
		**AIC**	**BIC**	**logLik**	**deviance**
		1836.1	1864.4	-911.1	1822.1

### Referent Selection Response Times

**Fig. 4:**
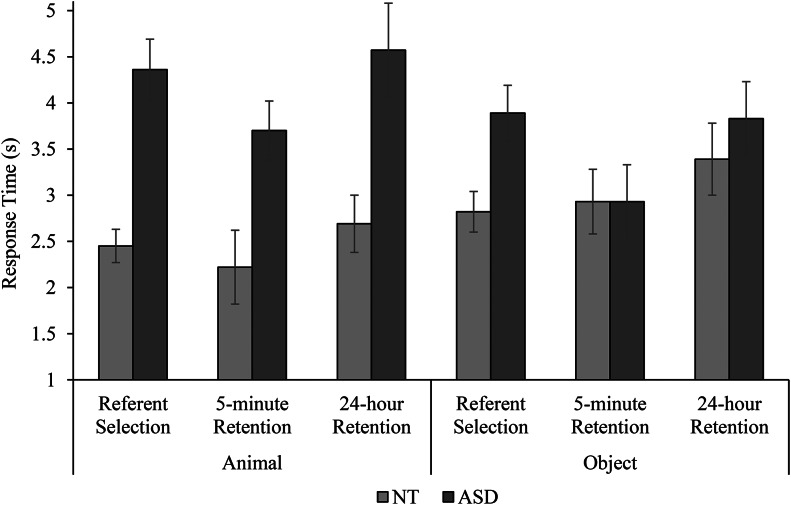
Mean response times on correctly answered referent selection, 5-minute retention, and 24-hour retention trials for neurotypical (NT) and autistic children (ASD), error bars show ± 1 *SE*

Children’s response times for correctly answered referent selection trials were analysed using linear mixed-effects models, testing the effects of population, condition, and trial type. We calculated the average correct response time for each population in each trial type and condition, and removed outliers that were ≥ 3*SD* above the mean for the sub-group (e.g. autistic children in the animal condition responding to novel trials). We also removed three trials from autistic children who did not use their hand to respond (e.g. they responded hand-over-hand, or using their head). The models in these analyses included 185 of 193 (96%) correct responses from autistic children, and 233 of 235 (99%) correct responses from neurotypical children. With outliers excluded, mean correct response times for each population are reported in Fig. 4.

The best fitting model included significant fixed effects of trial type (*t* = 4.40, *p* < .001), population (*t* = 2.19, *p* = .037) and a population x condition interaction (*t* = 2.65, *p* = .008; see Table 2). Children in both populations were slower to generate correct responses for novel trials than familiar trials. Autistic children took significantly longer than neurotypical children to respond correctly across both conditions, but the difference between groups was greater in the animal condition than in the object condition.

### 5-minute Retention Accuracy

Children’s retention accuracy after 5 minutes was analysed via generalised linear mixed-effects models testing the effects of population, condition, referent selection accuracy, novel referent selection trial accuracy, and number of repetitions of the target word heard at referent selection. We excluded nine trials for autistic participants and three trials for neurotypical participants due technical issues (8) and ambiguous responses (4). The models in these analyses contained 484 data points. The descriptive statistics are reported in Fig. 3.

The final model included a significant population x condition interaction (*z* = 2.94, *p* = .003; see Table 3). Autistic children responded significantly more accurately than neurotypical children in the animal condition (*z* = 2.50, *p* = .013), but not the object condition (*z* = -1.24, *p* = .22). Autistic children responded with significantly greater accuracy in the animal condition compared to the object condition (*z* = 2.08, *p* = .038), but neurotypical children did not significantly differ in their response accuracy between conditions (*z =* -1.69, *p* = .09).

**Table 3:** Summaries of the fixed effects in the final generalised linear mixed-effects models (log odds) of children’s accuracy on 5-minute retention trials

**Fixed effects**	**Estimated** **coefficient**	**Std. error**	***z***	**Pr(> |z|)**
(Intercept)	-0.32	0.15	-2.17	.03
Population	0.28	0.25	1.13	.26
Condition	0.12	0.21	0.59	.56
Population x Condition	1.24	0.42	2.94	.003
	**AIC**	**BIC**	**logLik**	**deviance**
	656.7	690.2	-320.4	640.7

### 5-minute Retention Response Times

Children’s response times for correctly answered 5-minute retention trials were analysed using linear mixed-effects models. Outliers were identified and removed in the same way as described for referent selection trials. The models in these analyses included 102 of 106 (96%) correct responses from autistic children and 99 of 100 (99%) correct responses from neurotypical children. With outliers excluded, mean correct response times for each population are reported in Fig. 4.

The inclusion of fixed effects (population and condition) did not improve model fit.

### 24-hour Retention Accuracy

Children’s retention accuracy after 24 hours was analysed via generalised linear mixed-effects models testing the effects of population, condition, referent selection accuracy, accuracy on novel referent selection trials, number of repetitions of the target word heard at referent selection, and total 5-minute retention accuracy (all coded as described previously). Two autistic children in the animal condition and one neurotypical child in the object condition did not complete the 24-hour retention trials due to absence. We excluded eight trials for autistic participants due to non-completion (1), ambiguous responses (4), and technical issues (3). The models in these analyses contained 464 data points. Descriptive statistics for 24-hour retention accuracy are presented in Fig. 3.

The best fitting model contained fixed effects of population (*z* = 1.92, *p* = .055), total accuracy at 5-minute retention (*z* = 4.43, *p* < .001), referent selection accuracy (*z* = 2.83, *p* = .005), and number of repetitions of the target word heard at referent selection (*z* = 3.18, *p* = .001; see Table 4). These results show that (1) autistic children responded more accurately than neurotypical children (marginally significant), (2) children with higher 5-minute retention accuracy were significantly more likely to respond correctly on 24-hour retention trials, (3) children who heard more repetitions of the target word at referent selection were significantly more likely to respond correctly after 24 hours, and (4) children with higher referent selection accuracy for individual novel words were significantly more likely to respond correctly at 24-hour retention. Note that the condition effect at 5-minute retention for autistic children was not detected after 24 hours.

**Table 4:** Summaries of the fixed effects in the final generalised and linear mixed-effects models (log odds) of children’s accuracy on 24-hour retention trials, and response times on correctly-answered 24-hour retention trials

	**Fixed effects**	**Estimated** **coefficient**	**Std. error**	***z***	**Pr(> |z|)**
Accuracy	(Intercept)	-2.14	0.44	-4.89	< .001
	Population	0.49	0.26	1.92	.055
	Total Accuracy at 5-minute Retention	0.35	0.08	4.43	< .001
	Referent Selection Accuracy	0.79	0.28	2.83	.005
	Number of Labels at Referent Selection	0.41	0.13	3.18	.001
		**AIC**	**BIC**	**logLik**	**deviance**
		604.8	642.0	-293.4	586.8
	**Fixed effects**	**Estimated coefficient**	**Std. error**	***t***	**Pr(> |t|)**
Response	(Intercept)	4.01	0.50	7.98	< .001
Times	Population	1.89	1.00	1.88	.072
	Condition	0.26	0.32	0.82	.41
	Population x Condition	1.81	0.64	2.82	.005
		**AIC**	**BIC**	**logLik**	**deviance**
		1106.1	1126.7	-547.1	1094.1

### 24-hour Retention Reaction Times

Children’s response times for correctly-answered 24-hour retention trials were analysed using linear mixed-effects models. Outliers were identified and removed in the same way as for previous analyses. These analyses included 121 of 128 (95%) correct responses from autistic children, and 107 of 109 (98%) correct responses from neurotypical children. With outliers excluded, mean correct response times for each population are reported in Fig. 4.

The best fitting model included a significant population x condition interaction (*t* = 2.82, *p* = .005; see Table 4). Autistic children took significantly longer than neurotypical children to respond correctly in the animal condition, but not in the object condition.

## Discussion

This study examined whether autistic and neurotypical children differ in their ability to disambiguate and retain novel words associated with high-interest and neutral-interest stimulus categories. Importantly, we examined children’s accuracy and response speed across three distinct stages of word learning: referent selection, 5-minute retention, and 24-hour retention after a period of sleep. In comparison to neurotypical controls matched on receptive vocabulary, autistic children did not significantly differ in accuracy when spontaneously disambiguating the meanings of novel words using ME across conditions. After 5 minutes, autistic children retained significantly more novel word-referent mappings for animal stimuli compared to object stimuli, whereas neurotypical children retained novel words for both animals and objects with comparable accuracy. Autistic children also retained more novel animal names after a 5-minute delay than neurotypical children. Surprisingly, after a 24-hour delay, autistic children retained novel word-referent mappings with greater accuracy than neurotypical children (marginally significant difference). However, autistic children demonstrated slower response times than neurotypical children at each word learning stage (with significant differences detected at referent selection and 24-hour retention).

As predicted, our participants’ referent selection across conditions demonstrates that both autistic and neurotypical children can employ ME to accurately identify the meanings of novel words, regardless of whether intended referents correspond with categories of interest. These findings, alongside existing evidence, show that autistic children can perform ME-based referent selection with similar accuracy to neurotypical children when expectations are based on receptive vocabulary (e.g. Carter & Hartley, 2021; Preissler & Carey, 2005). Using ME to actively disambiguate word meanings may represent a critical strategy through which both neurotypical and autistic children establish correct word-referent associations for neutral- and high-interest stimuli, increasing the quality of their audio-visual input and potentially contributing to long-term vocabulary development (Hartley et al., 2019). Unsurprisingly, children in both populations responded more accurately on familiar trials than novel trials as they had pre-existing representations of referents for requested words.

Also in support of our predictions, effects of stimulus condition were observed at 5-minute retention. Here, autistic children achieved significantly greater accuracy in the animal condition – where they surprisingly exceeded neurotypical children – than in the object condition. As autistic children tend to process high-interest stimuli with greater focus and intensity (Sasson et al., 2011), it may be that their interest in animals facilitated encoding of more robust word-referent representations that were less vulnerable to decay after five minutes. Indeed, previous studies have demonstrated positive relationships between children’s attentional focus during word-referent mapping and subsequent retention accuracy (Bion et al., 2013; Hilton et al., 2019). It is also well-documented that many autistic individuals are adept at memorising information associated with topics and events of heightened interest (Bölte & Poustka, 2008; Happé, 1999). By contrast, neurotypical children’s 5-minute retention accuracy did not significantly differ between conditions. These findings show that autistic children experience short-term retention benefits for words associated with high-interest stimuli.

In contrast to 5-minute retention, after 24 hours we observed that autistic children retained novel words for *both* objects and animals with greater accuracy than neurotypical children, and the condition effect on autistic children’s retention accuracy disappeared. For autistic children, overnight improvement in the object condition compared to the animal condition may be attributed to sleep having more beneficial consolidation effects on weaker memory representations (Drosopoulos et al., 2007; Williams & Horst, 2014). Sleep plays a critical role in protecting newly acquired declarative memories against decay, and many studies have demonstrated that neurotypical children retain words more accurately after sleep (e.g. Axelsson et al., 2018; Williams & Horst, 2014). Active system consolidation theory (Diekelmann & Born, 2010) posits that sleep enhances novel word retention through the reactivation of recently encoded word-referent representations. New word-referent representations are initially fragile, but reactivation during sleep facilitates their integration into memory networks enabling longer-term retention (Gais & Born, 2004). While limited evidence suggests that novel word retention in intellectually-able autistic children with age-expected language abilities may benefit from overnight sleep (e.g. Fletcher et al., 2020; Henderson et al., 2014), this study is the first to show a similar effect in autistic children with delayed language development.

One explanation for the observed between-population difference in 24-hour retention accuracy concerns chronological age. Children experience shorter sleep cycles than adults until 6 years (Hill et al., 2007; Montgomery-Downs et al., 2006), but longer sleep cycles are more beneficial for novel word consolidation (Diekelmann & Born, 2010). Therefore, it is possible that our autistic participants benefited more from overnight sleep because their average age exceeded 6 years, while the average age of the neurotypical children was significantly younger at just over 4 years. However, it is important to note that autism is commonly characterised by sleep disorders (e.g. bedtime resistance, sleep anxiety, difficulties falling asleep, parasomnia) that have the potential to negatively impact on overnight lexical consolidation and long-term vocabulary development (Díaz-Román et al., 2018; Souders et al., 2009). As no previous studies have tested 24-hour retention in autistic children with delayed language development, further research is required to replicate this effect and draw comparisons against neurotypical children matched on chronological age (in addition to children matched on receptive vocabulary) to control for developmental differences in sleep cycles. We also recommend that future studies investigate whether individual differences in sleep quality, duration, and disturbances predict variability in overnight consolidation of novel words for autistic children with language impairments.

At 24-hour retention, we found that both autistic and neurotypical children responded more accurately when they had heard more label repetitions during referent selection. This result highlights an important relationship between fast mapping and longer-term retention – *quantity* of auditory input received during referent selection influences the likelihood of successful memory consolidation. As proposed by the dynamic associative model (McMurray et al., 2012), successful identification of meaning may not necessarily support retention unless sufficient statistical input has been experienced. Cross-situational word learning studies show how more frequent exposures to word-referent pairings can increase children’s uptake from input and support encoding of word-referent representations that can be retrieved after delays (Hartley et al., 2020). Thus, for both autistic and neurotypical children, repeated exposures to novel word-referent associations may be critical to successful vocabulary acquisition, emphasising the importance of repetition as a component of communication interventions.

While response accuracy indicates whether children successfully identified and retained word-referent pairings, the time taken to generate correct responses provides insight into the speed of children’s information processing. At referent selection, children in both populations were quicker to respond correctly on familiar trials than novel trials. As children already knew the meanings of familiar words, correct responding simply required visual recognition of familiar referents. However, on hearing a novel word, children had to disambiguate the meaning of the word via mutual exclusivity. This required children to evaluate familiar competitors, ruling them out as targets, and shift their attention to the novel stimulus (Halberda, 2003). Since this task is more cognitively demanding, it is unsurprising that children were slower to make their selections on novel trials (Bion et al., 2013).

Critically, autistic children took significantly longer than younger neurotypical children to generate correct responses, particularly in the animal condition. This finding aligns with previous evidence (e.g. Hartley et al., 2020) and suggests that, although word learning mechanisms appear to be intact, autistic children may require longer to process audio-visual stimuli in the service of word learning. Delays in processing stimuli could be attributed to general learning difficulties or differences in visual attention disrupting children’s intake of information (Arunachalam & Luyster, 2018; Venker et al., 2018). On the other hand, autistic children’s particularly slow responses in the animal condition across test stages could be due to their heightened interest in the novel stimuli (i.e. they chose to spend longer studying items in the array before identifying referents). Longer response times at referent selection may have ultimately benefitted their subsequent retention accuracy by affording more time to encode each target’s perceptual features and providing the opportunity to hear more repetitions of the corresponding label. By extension, it is possible that neurotypical children’s retention accuracy would have increased if they had also taken longer to respond on referent selection trials. Thus, we recommend that future research investigates potential speed-accuracy trade-offs across word learning mechanisms in autism and neurotypical development.

Thinking practically, our findings have the potential to inform the development of interventions designed to scaffold autistic children’s word learning. While autistic children are often highly motivated to interact with touch-screen technology, evidence of effective learning via this platform has been mixed (Allen et al., 2016; Wainwright et al., 2020). Our study demonstrates that it is possible to teach children new words associated with different types of stimuli using a touch-screen computer when distractions are minimised. Additionally, we have shown that employing ME-based referent selection is an effective way to facilitate autistic children’s word learning. Presenting limited options helps children to utilise their existing vocabulary to engage in active learning, deciphering which novel referent is associated with a novel word. Furthermore, progression through trials was dependent on the speed of children’s responses, enabling them to engage with stimuli at their own pace. In natural environments, speech occurs at a rate of approximately 150 words-per-minute (Studdert-Kennedy, 1986), significantly faster than in most experimental contexts. The increased rate of stimuli presentation and greater attentional demands in natural communicative situations could create a processing bottleneck for autistic children, reducing the quality of their visual-auditory input and strength of associations between words and referents (Hartley et al., 2020; McMurray et al., 2012). As such, applying unrestricted processing times in clinical and educational interventions, as well as natural learning environments where possible, may facilitate autistic children’s vocabulary acquisition.

This study is not without limitations. Firstly, we must reflect on the implications of matching autistic and neurotypical children on receptive vocabulary, but not chronological age (the autistic sample was significantly older than the neurotypical sample). We selected these matching criteria because the study’s purpose was to compare word learning abilities across populations when delays in language development were controlled for. Previous studies comparing various aspects of language development in autism against chronological age norms for neurotypical children have consistently found deficits (e.g. Charman et al., 2003; Luyster et al., 2007). However, these differences could be due to various factors, including neurotypical children’s generally superior vocabulary learning abilities and differences in nonverbal intelligence. As such, matching on receptive vocabulary allows us to identify whether autistic children fundamentally differ in *how* they learn words relative to neurotypical children with similar vocabularies. Secondly, we acknowledge that our findings are derived from a single study with modest sample sizes. Unfortunately, our recruitment of participants was hindered by school closures and lockdown restrictions associated with the COVID-19 pandemic which occurred whilst the study was underway. Thus, we recommend that future studies seek to replicate our findings with larger samples.

In summary, this study has advanced understanding of how autistic and neurotypical children identify and retain novel word meanings, and how these processes are influenced by interests in stimulus categories. Despite our autistic participants’ delayed language development, they responded at least as accurately as vocabulary-matched neurotypical children on measures of referent selection, 5-minute retention, and 24-hour retention. Differences between neutral- and high-interest stimuli were only observed at 5-minute retention, where autistic children recalled animal names significantly more accurately than object names. This condition advantage disappeared after 24 hours, suggesting that superior learning of words associated with high-interest stimuli was relatively short-term. Thus, under favourable experimental conditions, differences in attention to stimuli that are perceived to be more or less interesting may not be detrimental to autistic children’s word learning. Although these results demonstrate that fundamental word learning mechanisms are not atypical in autism, autistic children were slower than neurotypical children to generate correct responses, particularly in the animal condition. As children responded at their own pace and processing times were unrestricted, spending longer studying stimuli may have benefited autistic children’s accuracy (i.e. a speed-accuracy trade-off). However, restricted processing times and the rapid pace of input during naturalistic communicative interactions could place strain on autistic children’s word learning mechanisms and impact on their accuracy. Our findings also indicate environmental conditions to scaffold word learning in clinical and educational contexts.

## References

[CR1] Ackermann, L., Hepach, R., & Mani, N. (2020). Children learn words easier when they are interested in the category to which the word belongs. *Developmental Science*, *23*(3), 10.1111/desc.12915. 10.1111/desc.12915 31618505

[CR2] Allen, M. L., Hartley, C., & Cain, K. (2016). iPads and the use of “apps” by children with autism spectrum disorder: Do they promote learning? *Frontiers in Psychology*, *7*, 1305. 10.3389/fpsyg.2016.01305. 27625621 10.3389/fpsyg.2016.01305 PMC5004059

[CR3] Artis, J., & Arunachalam, S. (2023). Semantic and Syntactic Properties of words and the receptive–expressive gap in autistic and non-autistic children. *Journal of Speech Language and Hearing Research*, 1–21. 10.1044/2023_JSLHR-22-00369. 10.1044/2023_JSLHR-22-00369 PMC10457093 37137280

[CR4] Arunachalam, S., & Luyster, R. (2018). Lexical Development in Young Children with Autism Spectrum disorder (ASD): How ASD May affect Intake from the Input. *Journal of Speech Language and Hearing Research*, *61*(11), 2659–2672. 10.1044/2018_JSLHR-L-RSAUT-18-0024. 10.1044/2018_JSLHR-L-RSAUT-18-0024 PMC6693575 30418494

[CR5] Axelsson, E., Churchley, K., & Horst, J. (2012). The right thing at the right time: Why ostensive naming facilitates word learning. *Frontiers in Psychology*, *3*, 88. 10.3389/fpsyg.2012.00088. 22470363 10.3389/fpsyg.2012.00088 PMC3314248

[CR6] Axelsson, E., Swinton, J., Winiger, A. I., & Horst, J. S. (2018). Napping and toddlers’ memory for fast-mapped words. *First Language*, *38*(6), 582–595. 10.1177/0142723718785490. 10.1177/0142723718785490

[CR7] Baron-Cohen, S., Baldwin, D. A., & Crowson, M. (1997). Do children with autism use the speaker’s direction of gaze strategy to crack the code of language? *Child Development*, *68*, 48–57. 10.2307/1131924. 9084124 10.2307/1131924

[CR8] Bates, D., Maechler, M., Bolker, B., & Walker, S. (2015). Lme4: Linear mixed-effects models using Eigen and S4. R package version 1.1–7. 2014 Institute for Statistics and Mathematics of WU website http://CRAN.R-project.org/package=lme4.

[CR9] Bion, R., Borovsky, A., & Fernald, A. (2013). Fast mapping, slow learning: Disambiguation of Novel Word-Object Mappings in Relation to Vocabulary Learning at 18, 24, and 30 months. *Cognition*, *126*(1), 39–53. 10.1016/j.cognition.2012.08.008. 23063233 10.1016/j.cognition.2012.08.008 PMC6590692

[CR10] Bölte, S., & Poustka, F. (2004). Comparing the profiles of savant and non-savant individuals with autistic disorder. *Intelligence*, *32*, 121–131. 10.1016/j.intell.2003.11.002. 10.1016/j.intell.2003.11.002

[CR11] Brown, H., Weighall, A., Henderson, L. M., & Gaskell, M. G. (2012). Enhanced recognition and recall of new words in 7- and 12-year-olds following a period of offline consolidation. *Journal of Experimental Child Psychology*, *112*, 56–72. 10.1016/j.jecp.2011.11.010. 22244988 10.1016/j.jecp.2011.11.010

[CR12] Carey, S., & Bartlett, E. (1978). Acquiring a single new word. *Papers and Reports on Child Language Development*, *15*, 17–29.

[CR13] Carpenter, M., Nagell, K., Tomasello, M., Butterworth, G., & Moore, C. (1998). Social cognition, joint attention, and communicative competence from 9 to 15 months of age. *Monographs of the Society of Research in Child Development*, *63*, 1–143. 10.2307/1166214. 10.2307/1166214 9835078

[CR14] Carter, C. K., & Hartley, C. (2021). Are children with autism more likely to retain object names when learning from colour photographs or black-and-white cartoons? *Journal of Autism and Developmental Disorders*, *51*(9), 3050–3062. 10.1007/s10803-020-04771-2. 33156474 10.1007/s10803-020-04771-2 PMC8349349

[CR15] Celani, G. (2002). Human beings, animals and inanimate objects: What do people with Autism Like? *Autism: The International Journal of Research and Practice*, *6*(1), 93–102. 10.1177/1362361302006001007. 11918112 10.1177/1362361302006001007

[CR16] Charman, T., Drew, A., Baird, C., & Baird, G. (2003). Measuring early language development in preschool children with autism spectrum disorder using the MacArthur Communicative Development Inventory (Infant Form). *Journal of Child Language*, *30*(1), 213–236. 10.1017/S0305000902005482. 12718299 10.1017/S0305000902005482

[CR17] Dale, P. S., & Fenson, L. (1996). Lexical development norms for young children. *Behavior Research Methods Instruments & Computers*, *28*, 125–127. 10.3758/BF03203646

[CR18] Díaz-Román, A., Zhang, J., Delorme, R., Beggiato, A., & Cortese, S. (2018). Sleep in youth with autism spectrum disorders: Systematic review and meta-analysis of subjective and objective studies. *Evidence-based mental health*, *21*(4), 146–154. 10.1136/ebmental-2018-300037. 30361331 10.1136/ebmental-2018-300037 PMC10270396

[CR19] Diekelmann, S., & Born, J. (2010). The memory function of sleep. *Nature Reviews Neuroscience*, *11*(2), 114–126. 10.1038/nrn2762. 20046194 10.1038/nrn2762

[CR20] Drosopoulos, S., Schulze, C., Fischer, S., & Born, J. (2007). Sleep’s function in the spontaneous recovery and consolidation of Memories. *Journal of Experimental Psychology*, *136*(2), 169–183. 10.1037/0096-3445.136.2.169. 17500644 10.1037/0096-3445.136.2.169

[CR21] Dunn, L. M., Dunn, L. M., Whetton, C., & Burley, J. (1997). *The british picture vocabulary scale* (2nd ed.). Windsor: NFER-Nelson.

[CR22] Elsabbagh, M., Volein, A., Holmboe, K., Tucker, L., Csibra, G., Baron-Cohen, S., & Johnson, M. H. (2009). Visual orienting in the early broader autism phenotype: Disengagement and facilitation. *Journal of Child Psychology and Psychiatry*, *50*, 637–642. 10.1111/j.1469-7610.2008.02051.x. 19298466 10.1111/j.1469-7610.2008.02051.x PMC3272379

[CR23] Fenson, L., Dale, P., Reznick, S. J., Bates, E., Thal, D. J., & Pethick, S. J. (1994). Variability in early communicative development. *Monographs of the Society for Research in Child Development*, *59*, 1–173. 10.2307/1166093. 7845413 10.2307/1166093

[CR24] Fletcher, F. E., Knowland, V., Walker, S., Gaskell, M. G., Norbury, C., & Henderson, L. M. (2020). Atypicalities in sleep and semantic consolidation in autism. *Developmental Science*, *23*(3), 10.1111/desc.12906. 10.1111/desc.12906 PMC7187235 31569286

[CR25] Friedrich, M., & Friederici, A. D. (2011). Word learning in 6-month-olds: Fast encoding – weak retention. *Journal of Cognitive Neuroscience*, *23*(11), 3228–3240. 10.1162/jocn_a_00002. 21391764 10.1162/jocn_a_00002

[CR26] Gais, S., & Born, J. (2004). Declarative memory consolidation: Mechanisms acting during human sleep. *Learning & Memory*, *11*(6), 679–685. 10.1101/lm.80504. 15576885 10.1101/lm.80504 PMC534696

[CR27] Gleitman, L. (1990). The structural sources of verb meanings. *Language Acquisition*, *1*(1), 3–55. 10.1207/s15327817la0101_2. 10.1207/s15327817la0101_2

[CR28] Halberda, J. (2003). The development of a word-learning strategy. *Cognition*, *87*(1), B23–B34. 10.1016/S0010-0277(02)00186. 12499109 10.1016/S0010-0277(02)00186

[CR29] Happé, F. (1999). Autism: Cognitive deficit or cognitive style? *Trends in Cognitive Sciences*, *3*, 216–222. 10.1016/S1364-6613(99)01318-2. 10354574 10.1016/S1364-6613(99)01318-2

[CR30] Hartley, C., Bird, L., & Monaghan, P. (2019). Investigating the relationship between fast mapping, retention, and generalisation of words in children with autism spectrum disorder and typical development. *Cognition*, *187*, 126–138. 10.1016/j.cognition.2019.03.001. 30861409 10.1016/j.cognition.2019.03.001

[CR31] Hartley, C., Bird, L., & Monaghan, P. (2020). Comparing cross-situational word learning, retention, and generalisation in children with autism and typical development. *Cognition*, *200*, 104265. 10.1016/j.cognition.2020.104265. 32259659 10.1016/j.cognition.2020.104265

[CR32] Henderson, L., Powell, A., Gaskell, G. M., & Norbury, C. (2014). Learning and consolidation of new spoken words in autism spectrum disorder. *Developmental Science*, *17*(6), 858–871. 10.1111/desc.12169. 24636285 10.1111/desc.12169

[CR33] Hill, C. M., Hogan, A. M., & Karmiloff-Smith, A. (2007). To sleep, perchance to enrich learning? *Archives Of Disease In Childhood*, *92*, 637–643. 10.1136/adc.2006.096156. 17588978 10.1136/adc.2006.096156 PMC2083752

[CR35] Hilton, M., & Westermann, G. (2017). The effect of shyness on children’s formation and retention of novel word–object mappings. *Journal of Child Language*, *44*(6), 1394–1412. 10.1017/S030500091600057X. 27916017 10.1017/S030500091600057X

[CR34] Hilton, M., Twomey, K. E., & Westermann, G. (2019). Taking their eye off the ball: How shyness affects children’s attention during word learning. *Journal of Experimental Child Psychology*, *183*, 134–145. 10.1016/j.jecp.2019.01.023. 30870698 10.1016/j.jecp.2019.01.023

[CR36] Horst, J. S., & Hout, M. C. (2016). The novel object and unusual name (NOUN) database: A collection of novel images for use in experimental research. *Behavior Research Methods*, *48*(4), 1393–1409. 10.3758/s13428-015-0647-3. 26424438 10.3758/s13428-015-0647-3

[CR37] Horst, J. S., & Samuelson, L. K. (2008). Fast Mapping but Poor Retention by 24-Month-Old Infants. *Infancy*, *13*(2), 128–157. 10.1080/15250000701795598. 33412722 10.1080/15250000701795598

[CR38] Kenny, L., Hattersley, C., Molins, B., Buckley, C., Povey, C., & Pellicano, E. (2016). Which terms should be used to describe autism? Perspectives from the UK autism community. *Autism: The International Journal of Research and Practice*, *20*(4), 442–462. 10.1177/1362361315588200. 26134030 10.1177/1362361315588200

[CR39] Kover, S., McDuffie, A. S., Hagerman, R. J., & Abbeduto, L. (2013). Receptive vocabulary in boys with Autism Spectrum Disorder: Cross-sectional developmental trajectories. *Journal of Autism and Developmental Disorders*, *43*(11), 2696–2709. 10.1007/s10803-013-1823-x. 23588510 10.1007/s10803-013-1823-x PMC3797266

[CR41] Leekam, S., Tandos, J., McConachie, H., Meins, E., Parkinson, K., Wright, C., Turner, M., Arnott, B., Vittorini, L., & Le Couteur, A. (2007). Repetitive behaviours in typically developing 2-year-olds. *Journal of Child Psychology and Psychiatry*, *48*(11), 1131–1138. 10.1111/j.1469-7610.2007.01778.x. 17995489 10.1111/j.1469-7610.2007.01778.x

[CR40] Leekam, S., Prior, M., & Uljarevic, M. (2011). Restricted and repetitive behaviors in Autism Spectrum Disorders: A review of Research in the last decade. *Psychological Bulletin*, *137*(4), 562–593. 10.1037/a0023341. 21574682 10.1037/a0023341

[CR43] Lord, C., Rutter, M., & Le Couteur, A. (1994). Autism Diagnostic interview – revised: A revised version of a diagnostic interview for caregivers of individuals with possible pervasive developmental disorders. *Journal of Autism and Developmental Disorders*, *24*(5), 659–685. 10.1007/BF02172145. 7814313 10.1007/BF02172145

[CR42] Lord, C., Rutter, M., DiLavore, P. C., & Risi, S. (2002). *Autism diagnostic observation schedule* (WPS ed.), Western Psychological Services, Los Angeles.

[CR45] Luyster, R., & Lord, C. (2009). Word learning in children with autism spectrum disorders. *Developmental psychology*, *45*(6), 1774–1786. 10.1037/a0016223. 19899931 10.1037/a0016223 PMC3035482

[CR44] Luyster, R., Lopez, K., & Lord, C. (2007). Characterizing communicative development in children referred for Autism Spectrum Disorders using the MacArthur-Bates Communicative Development Inventory (CDI). *Journal of Child Language*, *34*(3), 623–654. 10.1017/S0305000907008094. 17822142 10.1017/S0305000907008094

[CR46] Markman, E. M. (1989). Mutual exclusivity. In E. M. Markman (Ed.), *Categorization and naming in children: Problems of induction* (pp. 187–215). Cambridge, MA: MIT Press.

[CR47] Martin, F., & Farnum, J. (2002). Animal-assisted therapy for children with Pervasive Developmental Disorders. *Western Journal of Nursing Research*, *24*(6), 657–670. 10.1177/019394502320555403. 12365766 10.1177/019394502320555403

[CR48] McGregor, K. K., Rost, G., Arenas, R., Farris-Trimble, A., & Stiles, D. (2013). Children with ASD can use gaze in support of word recognition and learning. *Journal of Child Psychology and Psychiatry*, *54*(7), 745–753. 10.1111/jcpp.12073. 23574387 10.1111/jcpp.12073 PMC4232219

[CR49] McMurray, B., Horst, J., & Samuelson, L. (2012). Word Learning emerges from the Interaction of Online Referent selection and slow associative learning. *Psychological Review*, *119*(4), 831–877. 10.1037/a0029872. 23088341 10.1037/a0029872 PMC3632668

[CR50] Montgomery-Downs, H. E., O’Brien, L. M., Gulliver, T. E., & Gozal, D. (2006). Polysomnographic characteristics in normal preschool and early school-aged children. *Pediatrics*, *117*, 741–753. 10.1542/peds.2005-1067. 16510654 10.1542/peds.2005-1067

[CR51] Mullen, E. M. (1995). *Mullen scales of early learning*. Circle Pines, MN: American Guidance Service: AGS).

[CR52] Noterdaeme, M., Mildenberger, K., Minow, F., & Amorosa, H. (2002). Evaluation of neuromotor deficits in children with autism and children with a specific speech and language disorder. *European Child & Adolescent Psychiatry*, *11*(5), 219–225. 10.1007/s00787-002-0285-z. 12469239 10.1007/s00787-002-0285-z

[CR53] Ozonoff, S., Cook, I., Coon, H., Dawson, G., Joseph, R. M., Klin, A., & Wrathall, D. (2004). Performance on Cambridge Neuropsychological Test Automated Battery subtests sensitive to frontal lobe function in people with autistic disorder: Evidence from the Collaborative Programs of Excellence in Autism Network. *Journal of Autism and Developmental Disorders*, *34*(2), 139–150. 10.1023/B:JADD.0000022605.81989.cc. 15162933 10.1023/B:JADD.0000022605.81989.cc

[CR54] Pierce, K., Conant, D., Hazin, R., Stoner, R., & Desmond, J. (2011). Preference for geometric patterns early in life as a risk factor for autism. *Archives of General Psychiatry*, *68*(1), 101–109. 10.1001/archgenpsychiatry.2010.113. 20819977 10.1001/archgenpsychiatry.2010.113 PMC4894313

[CR55] Preissler, M., & Carey, S. (2005). The role of inferences about referential intent in word learning: Evidence from autism. *Cognition*, *97*(1), B13–B23. 10.1016/j.cognition.2005.01.008. 15925356 10.1016/j.cognition.2005.01.008

[CR56] Prothmann, A., Ettrich, C., & Prothmann, S. (2009). Preference for, and responsiveness to, people, dogs and objects in children with autism. *Anthrozoös*, *22*(2), 161–171. 10.2752/175303709X434185. 10.2752/175303709X434185

[CR57] Pupura, D. J., & Lonigan, C. J. (2009). Conners’ teacher rating scale for Preschool Children: A revised, brief, age-specific measure. *Journal of Clinical Child and Adolescent Psychology*, *38*(2), 263–272. 10.1080/15374410802698446. 19283604 10.1080/15374410802698446 PMC3279732

[CR58] Richler, J., Bishop, S. L., Kleinke, J. R., & Lord, C. (2007). Restricted and repetitive behaviors in young children with autism spectrum disorders. *Journal of Autism and Developmental Disorders*, *37*, 73–85. 10.1007/s10803-006-0332-6. 17195920 10.1007/s10803-006-0332-6

[CR59] Roid, G. H., Miller, L. J., Pomplun, M., & Koch, C. (2013). *Leiter international performance scale (Leiter-3)*. Los Angeles: Western Psychological Services.

[CR60] Samuelson, L., Kucker, S., & Spencer, J. (2017). Moving Word Learning to a Novel Space: A dynamic Systems View of Referent Selection and Retention. *Cognitive Science*, *41*(S1), 52–72. 10.1111/cogs.12369. 27127009 10.1111/cogs.12369 PMC5086318

[CR61] Sasson, N. J., Elison, J. T., Turner-Brown, L. M., Dichter, G. S., & Bodfish, J. W. (2011). Brief report: Circumscribed attention in young children with autism. *Journal of Autism and Developmental Disorders*, *41*, 242–247. 10.1007/s10803-010-1038-3. 20499147 10.1007/s10803-010-1038-3 PMC3709851

[CR62] Schopler, E., Van Bourgondien, M. E., Wellman, G. J., & Love, S. R. (2010). *Childhood autism rating scale CARS-2* (2nd ed.). Los Angeles: Western Psychological Services.

[CR63] Souders, M. C., Mason, T. B., Valladares, O., Bucan, M., Levy, S. E., Mandell, D. S., Weaver, T. E., & Pinto-Martin, J. (2009). Sleep behaviors and sleep quality in children with autism spectrum disorders. *Sleep*, *32*(12), 1566–1578. 10.1093/sleep/32.12.1566. 20041592 10.1093/sleep/32.12.1566 PMC2786040

[CR64] Studdert-Kennedy, M. (1986). *Some developments in research on language behavior* N.J. In D. R. Smelser, & Gerstein (Eds.), *Behavioral and social science: Fifty years of discovery: In commemoration of the fiftieth anniversary of the “Ogburn report: Recent social trends in the United States* (pp. 208–248). Washington, DC: National Academy Press.

[CR65] Tenenbaum, E. J., Amso, D., Righi, G., & Sheinkopf, S. J. (2017). Attempting to “Increase intake from the Input”: Attention and Word Learning in Children with Autism. *Journal of Autism and Developmental Disorders*, *47*, 1791–1805. 10.1007/s10803-017-3098-0. 28342164 10.1007/s10803-017-3098-0 PMC7916990

[CR66] Twomey, K. E., Morse, A. F., Cangelosi, A., & Horst, J. S. (2016). Children’s referent selection and word learning: Insights from a developmental robotic system. *Interaction Studies*, *17*(1), 101–127. 10.1075/is.17.1.05two. 10.1075/is.17.1.05two

[CR67] Venker, C., Bean, A., & Kover, S. (2018). Auditory–visual misalignment: A theoretical perspective on vocabulary delays in children with ASD. *Autism Research*, *11*(12), 1621–1628. 10.1002/aur.2038. 30475450 10.1002/aur.2038 PMC6871516

[CR68] Vlach, H. A., & Sandhofer, C. M. (2012). Fast mapping across time?: Memory processes support children’s retention of learned words. *Frontiers in Psychology*, *3*, 1–8. 10.3389/fpsyg.2012.00046. 22375132 10.3389/fpsyg.2012.00046 PMC3286766

[CR69] Wainwright, B., Allen, M. L., & Cain, K. (2020). Symbolic understanding and word–picture–referent mapping from iPads in Autism Spectrum Condition: The Roles of Iconicity and Engagement. *Journal of Autism and Developmental Disorders*, *50*(8), 2941–2956. 10.1007/s10803-020-04404-8. 32036494 10.1007/s10803-020-04404-8 PMC7374469

[CR70] Williams, K. T. (2007). *Expressive Vocabulary Test* 2nd Edn. Minneapolis, MN: Pearson Assessments.

[CR71] Williams, S. E., & Horst, J. S. (2014). Goodnight book: Sleep consolidation improves word-learning via storybooks. *Frontiers in Psychology*, *5*, 184. 10.3389/fpsyg.2014.00184. 24624111 10.3389/fpsyg.2014.00184 PMC3941071

